# Current and Future Directions of Stem Cell Therapy for Bladder Dysfunction

**DOI:** 10.1007/s12015-019-09922-2

**Published:** 2019-11-22

**Authors:** Jung Hyun Shin, Chae-Min Ryu, Hwan Yeul Yu, Dong-Myung Shin, Myung-Soo Choo

**Affiliations:** 1 Deparatment of Urology, Asan Medical Center, University of Ulsan College of Medicine, 88 Olympic-ro 43-gil, Songpa-gu, Seoul, 05505 South Korea; 2 Department of Biomedical Sciences, Asan Medical Center, University of Ulsan College of Medicine, 88 Olympic-ro 43-gil, Songpa-gu, Seoul, 05505 South Korea

**Keywords:** Stem cell therapy, Bladder dysfunction, Stress urinary incontinence, Overactive bladder, Detrusor underactivity, Interstitial cystitis

## Abstract

Stem cells are capable of self-renewal and differentiation into a range of cell types and promote the release of chemokines and progenitor cells necessary for tissue regeneration. Mesenchymal stem cells are multipotent progenitor cells with enhanced proliferation and differentiation capabilities and less tumorigenicity than conventional adult stem cells; these cells are also easier to acquire. Bladder dysfunction is often chronic in nature with limited treatment modalities due to its undetermined pathophysiology. Most treatments focus on symptom alleviation rather than pathognomonic changes repair. The potential of stem cell therapy for bladder dysfunction has been reported in preclinical models for stress urinary incontinence, overactive bladder, detrusor underactivity, and interstitial cystitis/bladder pain syndrome. Despite these findings, however, stem cell therapy is not yet available for clinical use. Only one pilot study on detrusor underactivity and a handful of clinical trials on stress urinary incontinence have reported the effects of stem cell treatment. This limitation may be due to stem cell function loss following ex vivo expansion, poor in vivo engraftment or survival after transplantation, or a lack of understanding of the precise mechanisms of action underlying therapeutic outcomes and in vivo behavior of stem cells administered to target organs. Efficacy comparisons with existing treatment modalities are also needed for the successful clinical application of stem cell therapies. This review describes the current status of stem cell research on treating bladder dysfunction and suggests future directions to facilitate clinical applications of this promising treatment modality, particularly for bladder dysfunction.

## Introduction

Stem cells can differentiate into a range of cell types, with a characteristic capacity for continuous self-renewal. These cells are thereby self-sustaining and can differentiate into progenitor cells to replace aging cells undergoing apoptosis [1, 2]. This potential has rendered stem cell research a predominant area of interest in the field of regenerative medicine, which focuses on fundamental over conservative treatments [3–5].

Stem cells are classified by their developmental capacity. Pluripotent stem cells (PSCs) can differentiate into all three germinal layers (endoderm, ectoderm, and mesoderm) and originate from the inner cell mass (ICM) of blastocysts during embryonic development. PSCs from blastocyst ICMs can be expanded ex vivo into immortalized embryonic stem cell (ESCs) lines [6, 7]. Patient-specific PSCs can be established either by somatic cell nuclear transfer (SCNT) or therapeutic cloning [8], or by cellular reprogramming using key transcription factors, including *Oct4*, *Sox2*, *Klf4*, and *Myc*, and are referred to as induced PSCs (iPSCs) [9–11]. PSCs, including ESCs, SCNT cells, and iPSCs, are the most important stem cell populations in regenerative medicine based on their pluripotency and capacity for unlimited expansion; however, safety concerns over malignant transformation or teratoma formation have limited their clinical use.

Adult tissues contain certain types of multipotent stem cells, such as hematopoietic and neural stem cells, able to differentiate into a specific cell lineage [12]. Among them, mesenchymal stem cells (MSCs) are multipotent progenitors that can differentiate into bone, cartilage, muscle, vascular smooth muscle cells, and other connective tissues [13, 14]. MSCs can be derived from a range of adult tissues, including bone marrow, adipose, peripheral blood, and dental pulp, among others, and fetal tissues including umbilical cord blood, Wharton’s jelly, placenta, and amniotic fluid. MSCs have several advantages over other types of stem cells, with fewer concerns over tumorigenicity than for PSCs, prominent availability from many different adult tissues, and enhanced proliferation and differentiation capacity over conventional adult stem cells. MSCs directly differentiate into the damaged tissue in which they are implanted. In addition, the cytokines and growth factors secreted by MSCs yield beneficial outcomes, called paracrine effects, by alleviating inflammatory reactions or tissue fibrosis, and stimulating angiogenesis and endogenous progenitor cells. These paracrine effects are regarded as the primary therapeutic mode of action for these cells [15]. MSCs can also migrate to damaged tissue in response to microenvironmental factors related to inflammation and ischemia gradients leading to damaged target tissues [16].

The use of stem cells in medicine began with bone marrow transplantation for myeloproliferative diseases. Stem cell therapy for other disease entities, including autoimmune disease, spinal cord injury, and cardiovascular disease have also been, and continue to be, investigated [4, 5, 17–20]. Bladder dysfunction is an excellent candidate for stem cell therapy due to its chronic nature and high prevalence [21, 22]. Current treatment modalities are suboptimal regardless of the underlying cause for bladder dysfunction, and this causes a significant loss in patients’ quality of life. Various preclinical models have been developed to evaluate the therapeutic effects of stem cells for bladder dysfunction, such as stress urinary incontinence, overactive bladder (OAB), detrusor underactivity (DUA), and interstitial cystitis/bladder pain syndrome (IC/BPS), but clinical studies in humans remain scarce. This report reviews current and future directions of stem cell research therapy for various types of bladder dysfunction.

## Stress Urinary Incontinence

Urinary incontinence or involuntary loss of urine is symptomatic terminology for various types of dysfunction, including stress urinary incontinence (involuntary leakage during exertion, sneezing, or coughing), urgency urinary incontinence (involuntary leakage associated with urgency), and mixed urinary incontinence wherein both of these are present. Stress urinary incontinence (SUI) is common in females, with an estimated prevalence between 29 and 75%. The pathophysiology of female SUI is associated with urethral hypermobility and intrinsic sphincter deficiency [23]. Conservative therapies include weight loss, Kegel exercises, and bio-feedback, which have been shown to be beneficial but require patient compliance. Surgical treatments are also employed, such as bladder neck suspension, pubovaginal sling, or mid-urethral sling, to reinforce periurethral support. Urethral injection therapy and artificial urethral sphincter have also been suggested for female SUI, but both have limited efficacy and little supporting evidence [24]. In male patients, SUI usually results from neurovascular bundle injury during radical prostatectomy or external urethral sphincter injury during transurethral resection or enucleation of the prostate. Depending on incontinence severity (number of pads used per day), behavioral therapy such as Kegel exercises and bio-feedback, pharmacotherapy, and surgical therapy, such as urethral bulking agent injection, male sling, or artificial urethral sphincter, are all treatment options. Each modality has proven efficacy but is accompanied by certain limitations. Artificial urethral sphincter is highly effective for severe male incontinence, but the device has a limited period of use, risk of infection, and time-dependent risks for urethral erosion and urethral atrophy. The inadequacy of existing treatments has led researchers to focus on stem cell therapy to improve or restore urethral sphincter function.

SUI animal models consist of two types, reversible incontinence, and durable incontinence. The first preclinical incontinence model was developed by Lin et al. in 1998, accomplished by inducing vaginal distension (VD) in female rats with a 2 mL, saline-filled intravaginal balloon. This VD model demonstrated significantly decreased periurethral muscle function and injured neurons in the pelvic plexus [25]. Urethral dysfunction is temporary in the VD model and mimics SUI in human females following vaginal delivery. A reversible incontinence model can also be generated with pudendal nerve crush; this nerve controls the external urethral sphincter and is often compressed during vaginal delivery. Electromyograms of pudendal nerve crush models showed denervation patterns followed by regeneration [26]. Durable incontinence models include direct or indirect periurethral sphincter injury, urethrolysis, electrocauterization, pubourethral ligament injury, and complete unilateral or bilateral pudendal nerve transection [27].

Autologous adipose-derived stem cells (ADSC), muscle-derived stem cells, muscular precursor cells, MSCs, and human MSCs have been used in preclinical studies evaluating stem cell therapy for urethral sphincter regeneration [28]. All stem cell types were shown to differentiate into myocyte-lineage stem cells upon injection, and all secreted substances that restored urethral sphincter function via paracrine effects.

Among different types of bladder dysfunction, SUI is the most researched in stem cell clinical trials [29–43]. These trials can be classified based on patient population (female or male) or type of stem cells used, such as muscle-derived stem cells (MDSCs) or non-muscle derived stem cells. Some study groups have demonstrated the therapeutic efficacy and safety of different types of stem cells in SUI treatment [44]. The first of these, using stem cell therapy for female SUI, was reported by Carr et al. [31]. Carr and colleagues injected autologous, myocyte-derived stem cells into the urethral sphincters of eight female SUI patients for whom previous non-invasive treatments had failed. At 12-month follow-up, six patients showed symptom improvement in pad tests, voiding diary, and quality of life questionnaires, including one patient that achieved complete continence [31]. In 2013, the trial was expanded to 38 patients and different stem cell dosages (1, 2, 4, 8, 16, 32, 64, or 128 × 10^6^). Patients treated with higher doses (≥ 32 × 10^6^) showed more symptom improvement those who received lower ones (≤ 16 × 10^6^) [32]. Other clinical trials on female SUI patients have used heterogeneous patient cohorts (age range 5–80) with mainly 12 months follow-up period.

The first clinical trial for male SUI was performed by Mitterberger et al. [40]. Mitterberger and colleagues injected autologous fibroblasts and myoblasts into the urethral submucosa and rhabdosphincter in 63 post-prostatectomy incontinence patients [40]. Gerullis et al. reported on the largest clinical trial therein on 222 males receiving five injections of autologous MDSCs around the rhabdosphincter [41]. Gotoh et al. injected autologous ADSCs into the external sphincters of male SUI patients who had undergone radical prostatectomy or transurethral resection of the prostate with Holmium laser, and therapeutic effects and safety were observed at both one-year and long-term follow-up [33, 45]. However, a consensus on the most suitable source and injection route of stem cells for SUI remains elusive. In addition, the numbers of clinical trials and of patients in current trials are small and the amount of evidence is limited, so the indication for stem cell therapy have yet to be agreed upon.

## Overactive Bladder (OAB)

Overactive bladder (OAB) is a chronic condition defined by urgency accompanied by increased frequency, nocturia, and urgency urinary incontinence. OAB can be due to a range of etiologies that include idiopathic, age-related, neurological, bladder outlet obstruction (BOO), and many others. Prevalence of OAB increases with age, and current treatments are behavioral, pharmacological, and surgical therapies. Antimuscarinics are most commonly prescribed for OAB, but their therapeutic effect is limited and accompanied by a high incidence of side-effects, such as dry mouth, constipation, and cognitive dysfunction, that lead to reduced persistency in these patients [46, 47]. The pathophysiology of OAB remains controversial; several causes, including myogenic, urotheliogenic (urothelium/suburothelium), urethrogenic, supraspinal, and detrusor underactivity have been suggested, and certain cofactors such as metabolic syndrome, sex hormone deficiency, affective or psychological disorders, functional gastrointestinal abnormalities, subclinical nervous system dysfunction, and urinary microbiota, have been implicated, but a consensus therein has yet to be reached [48].

Different conditions are used to induce OAB in animal models, such as chronic ischemia (vessel ligation or hyperlipidemia), spinal cord injury, early diabetes, and BOO [49]. Huang et al. induced hyperlipidemia in rats fed a high fat diet and then injected ADSCs into their tail veins [50]. Authors reported that ADSC treatment relieved OAB symptoms in a hyperlipidemia rat model by enhancing the microvessel and neuronal environment in the detrusor. Liang et al. used middle cerebral artery occlusion in female rats to induce detrusor overactivity with significant decreases in voided volume and intercontraction interval. Human amniotic fluid stem cells (hAFSCs) ameliorated this bladder dysfunction and were associated with increased expression of nerve growth factor (NGF), M2, M3 muscarinic, and P2X1 purinergic receptors [51]. Liang’s group then induced detrusor overactivity in female rats using arterial balloon injury of the common iliac artery with a 2Fr Fogarty balloon catheter and high cholesterol diet and found that hAFSCs injected into the tail vein improved histological abnormalities and voiding parameters by downregulation of oxidative stress inducers and TNF-α expression [52].

The majority of studies, however, employ BOO rat models. Woo et al. induced BOO in mice and then intravenously injected them with human umbilical cord-derived stem cells [53]. Treatment outcomes were evaluated 4 weeks after injection. The control BOO group presented with detrusor hypertrophy and collagen deposits, while the stem cell group had similar levels of smooth muscle hypertrophy and fibrosis as the negative control group. A two-fold increase in expression of C-C motif chemokine ligand-2 (CCL2) was observed in the stem cell group, and Woo and colleagues presumed that the recruitment potency of their stem cells was associated with CCL2. Lee et al. evaluated whether human mesoderm-derived stem cells could inhibit collagen deposition in a rat BOO model, and reported that the stem cell group presented with reductions in transforming growth factor-beta (TGF-β); his team concluded that the stem cells restored bladder function by inhibiting bladder fibrosis [54]. Song et al., induced BOO in six-week-old female rats; four weeks later, rats were injected with intravenous solifenacin, an anticholinergic frequently prescribed for bladder dysfunction in humans, daily for two weeks or green fluorescent protein (GFP)-labeled adipose-tissue derived human MSCs (AD-MSCs) once directly into the bladder. Song’s group reported that AD-MSCs alleviated bladder dysfunction via paracrine effects [55]. However, few transplanted AD-MSCs were detected via microscopy to evaluate GFP staining, and no RNA or DNA sequences were observed in any of the bladder tissue cells, indicating little assimilation of the administrated stem cells. Bladder tissues transplanted with AD-MSCs were instead characterized by an increase in rat sequence-specific genes transcription related to primitive PSCs such as *Oct4*, *Sox2*, and *Stella*, and stem cell trafficking, such as stromal cell-derived factor-1 (SDF-1), hepatocyte growth factor (HGF), platelet-derived growth factor (PDGF), vascular endothelial growth factor (VEGF), and their corresponding receptors [55]. These results suggest that transplanted AD-MSCs might stimulate the release and mobilization of *Oct4*^*+*^ primitive endogenous stem cells to the damaged bladder, indicating an inducement of paracrine effects by these stem cells.

## Detrusor Underactivity (DUA)

Detrusor underactivity (DUA) is a urodynamic diagnosis defined by the International Continence Society as “a contraction of reduced strength and/or duration, resulting in prolonged bladder emptying and/or failure to achieve complete bladder emptying within a normal time span.” DUA is estimated to affect between 12 to 45% of females and 9 to 28% of males under 50, a level that increases to approximately 48% in males over 70. Risk factors include aging, neurological deficits, diabetes mellitus, and persistent BOO. The pathophysiology of DUA has yet to be determined, and a range of causes such as idiopathic, changes in afferent or efferent neural control, or myogenic via degeneration of detrusor smooth muscle, or changes in ion exchange and transport has been suggested. No definitive treatment for DUA is indicated. Cholinergic pharmacotherapy has limited efficacy, and current therapeutic modalities include assisting bladder emptying with intermittent catheterization or an indwelling Foley catheter, or suprapubic cystostomy [56–58].

Among the different DUA models, the diabetic mouse model is most commonly used as it requires just a single intraperitoneal injection of streptozotocin (STZ) with or without a high-cholesterol diet. Animal DUA models can also be induced via chronic ischemia (vessel ligation or hyperlipidemia), pelvic nerve or spinal cord injury (direct trauma or cryoinjury), cerebral ischemia, diabetes, and persistent BOO. Considering these same techniques are also used to induce OAB in animal models, functional evaluations thereof are essential [49]. The different animal models for OAB or DUA and results of preclinical stem cell therapy on these models are summarized in Table 1.

**Table 1:** Stem cell therapy in preclinical models of OAB and DUA

Method	Animal/Sex	Reference	Model type	Cell type	Route of injection	Cell count (/mL)	Evaluation (days)	Mode of action
Bilateral iliac artery ischemia	SD Rat ♀	Neurourol Urodyn 2018 [52]	OAB	hAFSCs (human amniotic fluid-derived stem cells)	Intravenous tail injection	1.0 × 10^6^ /0.3 mL	1 3 7	TNFα
Cerebral ischemia	SD Rat ♀	Stem Cells Transl Med 2017 [51]	OAB	hAFSCs (human amniotic fluid-derived stem cells)	Bladder injection	1.0 × 10^6^ /0.3 mL	3 10	NGF, M2, M3, P2X1
Cerebral ischemia	SD Rat ♀	Taiwan J Obstet Gynecol 2016 [76]	OAB	hUCB-derived CD34^+^ (Umbilical cord)	Intravenous tail injection	1.0 × 10^6^ /0.3 mL	1 3 7	NGF, M2, M3
Bilateral iliac artery ischemia	SD Rat ♀	Int J Mol Med 2012 [77]	OAB	Rat BM-MSCs (Bone marrow)	iliac artery	4.0 × 10^6^ /tube +doxazosin mesylate I.P injection	56	regenerates bladder tissue
Partial bladder outlet obstruction (pBOO)	SD Rat ♀	J Pediatr Urol 2019 [78]	N/A	Rat BM-MSCs (Bone marrow)	Intravenous tail injection	1.0 × 10^6^ /0.5 mL	14 28	Anti-inflammatory, anti-fibrotic
Partial bladder outlet obstruction (pBOO)	SD Rat ♀	Can Urol Assoc J 2016 [79]	N/A	Rat MSCs	Intravenous tail injection	5.0 × 10^6^ /0.5 mL	7 14	Anti-inflammatory
Bladder outflow obstruction (BOO)	Lewis Rat ♂	World J Urol 2014 [80]	DUA	Rat ADSCs (Adipose-derived stem cells), Rat MPCs (muscle precursor cells)	Bladder injection	1.5 × 10^6^ /0.5 mL	42	regenerates bladder tissue
Bladder outflow obstruction (BOO)	SD Rat ♀	Stem Cells Dev 2014 [55]	OAB	hAD-MSCs (adipose-derived MSCs)	Bladder injection		28	SDF-1, HGF, paracrine effects
Partial bladder outlet obstruction (pBOO)	SD Rat ♀	Int J Urol 2014 [81]	OAB	Rat BM-MSCs (Bone marrow)	Intravenous tail injection	5.0 × 10^6^	42	morphological changes
Bladder outflow obstruction (BOO)	SD Rat ♀	Cell Transplant 2012 [54]		B10 human MSCs overexpress-ing HGF	Bladder injection	1.0 × 10^6^	28	HGF, TGF-β
Nerve injury	SD Rat ♀	Transplantation 2010 [82]	DUA	Sk-MSC (skeletal muscle-derived multipotent stem cells)	Damaged nerve lesion	5-7 × 10^5^	28	Pericytes Fibroblasts
Nerve injury	SD Rat ♀	Urology 2005 [83]	DUA	MDCs (Muscle-derived Cell)	Damaged nerve lesion	3 × 10^5^	14	Autograft
Cryo injury	NIH-rNu Rat ♀	Journal of Urology 2007 [84]	N/A	AF-MSC and BM-MSC	Bladder injection	2 × 10^6^	30	SMC TGFβ
Cryo injury	SCID mice and SD Rat ♀	Gene Therapy 2002 [85]	DUA	Muscle-derived cell	Bladder injection	1.5 × 10^6^	7 14 28 56	β-galactosidase AChRs
Cryo injury	C57BL/6 mice ♂	Journal of Urology 2009 [86]	DUA	Adipocyte derived fat cell	Bladder injection	5 × 10^4^	14 30	SMC TGFβ
Diabetes	SD Rat ♀	Stem Cells and Development 2012 [59]	OAB/DUA	ADSCs (adipose tissue-derived stem cells)	tail vein injection	3 × 10^6^	28	Apoptosis vascular integrity
Diabetes	SD Rat ♀	Sci Rep 2018 [62]	DUA	hAFSCs (human amniotic fluid stem cells)	Bladder injection	3 × 10^6^	28 84	NGF M2, M3 receptors

Zhang et al. were the first to report therapeutic efficacy of autologous AD-MSCs; the group induced type 2 diabetes in 8-week-old female rats with low-dose intraperitoneal STZ and a high fat diet to generate a diabetic bladder dysfunction rat model [59]. Experimental AD-MSCs were treated with 10 μM 5-ethynyl-2′-deoxyuridine (EdU) fluorescent dye for tracking and then injected via tail vein or directly into the bladder. After bladder injection, EdU-labelled AD-MSCs were predominantly visualized in the submucosal layer, with some EdU-positive nuclei appearing within smooth muscle actin-expressing cells. Intravenous injection of AD-MSCs ameliorated voiding dysfunction by direct differentiation into detrusor smooth muscle cells and paracrine effects that inhibited apoptosis and promoted angiogenesis. However, only 40 to 60% of the diabetic animals in the stem cell injection group showed voiding improvements. The same study group then added defocused low-energy shock wave (DLSW) to the protocol to maximize the therapeutic effect of AD-MSCs [60, 61]. In a preliminary study, neonatal rats received intraperitoneal EdU, and type 1 diabetes was induced with intraperitoneal STZ injection (60 mg/kg) four weeks later. Slow wave therapy restored damaged bladder function and improved angiogenesis and smooth muscle regeneration by activating endogenous stem cells and inducing them to secrete VEGF and NGF growth factors. Next, Zhang and colleagues compared the effects of autologous AD-MSCs versus AD-MSCs pretreated with DLSW in the same diabetic rat model. DLSW-pretreated AD-MSC showed greater efficacy and more secretion of VEGF and NGF. Recruitment of endogenous stem cells and associated growth factors were considered important findings from these studies.

Liang et al. also used an STZ-induced diabetic rat model to evaluate the effects of hAFSCs [62]. Liang’s group measured the immunoreactivities and mRNAs of NGF and M2 and M3 muscarinic receptors. Bladder dysfunction in STZ rats was improved by hAFSCs with recovery of NGF and muscarinic receptors suggested as a potential mechanism for the improvement. Therapeutic effects of different stem cells have been proved in other types of DUA models, as well.

In 2015, Levanovich et al. reported a pilot study on intra-detrusor injection of adult muscle-derived cells (AMDC) in a human DUA patient [63], a 79-year-old man who had twice undergone transurethral resection of the prostate and suffered from recurrent urinary retention despite the administration of alpha blockers and bethanechol. The patient was on clean intermittent catheterization 4 to 6 times per day but had repeated episodes of gross hematuria and febrile urinary tract infections. Approximately 150 mg of quadriceps femoris muscle was collected, and a final concentration of 250 million AMDCs was harvested. Under local anesthesia, AMDC was injected at a 2 mm depth using direct visualization with a flexible cystoscope. Each injection volume was 0.5 mL, with a total volume of 15 mL. The patient’s subjective symptoms improved, and the ability to void small volumes at 3 months post-treatment was observed, but the patient still required intermittent catheterization over the one-year follow-up period.

## Interstitial Cystitis/Bladder Pain Syndrome (IC/BPS)

Interstitial cystitis/bladder pain syndrome (IC/BPS) presents as chronic pelvic pain usually accompanied by frequency, nocturia, or urgency not associated with evidence of identifiable pathology [64]. IC/BPS predominantly occurs in women (M:F, 1:9) and is overall uncommon. In South Korea, IC/BPS prevalence was theorized to be 0.26% in 2011. Worldwide prevalence varies among studies, but recent evidence suggests that IC/BPS prevalence might be 2% or more in the female population. In addition to pain and voiding dysfunction, other IC/BPS symptoms include sexual dysfunction, sleep disturbance, anxiety, depression, and chronic stress, leading to significantly decreased quality of life. The pathophysiology of the syndrome remains controversial, but hypotheses include defects in the glycosaminoglycan (GAG) layer, disruption of urothelium permeability, autoimmunity, infection, and inflammation. The same treatment modalities described for other bladder dysfunction types, behavioral modification, pharmacological therapy, intravesical instillation, and cystoscopic surgery, have been proposed, but most are reported to provide only temporary symptom control and carry a high risk of recurrence.

Many investigations to identify the pathophysiology and delineate a treatment strategy for IC/BPS have been performed across a range of animal models. Animal modelling of IC/BPS includes the bladder-centric model, induced by the addition of toxic substances to the urine, and models with more complex mechanisms, such as modulation of bladder function through alterations to the central nervous system and psychological and physical stress induction [65]. No on-going clinical trials on stem cell therapy in IC/BPS are currently registered at clinicaltrial.gov. Eleven have been published that evaluated the therapeutic effects of stem cell treatments in an IC/BPS rat model, from South Korea (5), China (3), Japan (2), and Taiwan (1) (Table 2). All of these studies utilized bladder-centric rat models generated by intravesical instillation with chemical substances to induce the clinical characteristics of IC/BPS, which are urothelial denudation, inflammation, and bladder fibrosis with frequent micturition and small micturition volumes.

**Table 2:** Stem cell therapy in preclinical models of IC/BPS

Journal publication	Animal/Sex	Model induction	Stem cell type	Route of injection	Cell count (/mL)	Evaluation (days)	Mode of action
Theranostics 2018 [74]	SD				1.0 × 10^6^		
	Rat	PS/LPS	hESC-MSC	Bladder injection	0.5 × 10^6^	7	Wnt
	♀				0.25 × 10^6^	14	IGF
						28	
Int Neurourol J 2018 [72]	SD				1.0 × 10^6^		
	Rat	Ketamine	hESC-MSC	Bladder injection	0.5 × 10^6^	7	Anti-Fibrosis
	♀				0.25 × 10^6^		
Biochem Biophys Res Commun 2018 [69]							
	SD		hUCB-MSCs (Umbilical cord)				AKT
	Rat	CYP		Tail injection	1.0 × 10^6^	7	mTOR
	♀						
Int Urogynecol J 2018 [87]					1.0 × 10^6^/20 μL		
	SD	HCL	AD-MSCs (adipose tissue)	Bladder injection			TNFα
	Rat					14	TGFβ
	♀						VEGF
Sci Rep 2017 [70]	SD				1.0 × 10^6^	7	
	Rat	PS/LPS	hESC-MSC	Bladder injection	0.5 × 10^6^	14	Wnt
	♀				0.25 × 10^6^	28	
Stem Cell Res Ther 2017 [88]	SD		hUSCs (human urine)	Bladder instillation	1.2 × 10^6^/0.2 mL		Anti-inflammatory
	Rat	PS/LPS				5	
	♀						
Am J Transl Res 2017 [68]	SD	PS	BM-MSCs (Bone marrow)	Bladder transplan-tation, intraperitoneal injection	2 × 10^5^	30	TGF-β/MAPK
	Rat						
	♂/♀						
Sci Rep 2016 [71]	SD	Ketamine	hUCB-MSC (Umbilical cord)	Bladder injection	1.0 × 10^6^	7	Anti-fibrotic
	Rat						
	♀						
Cell Transplant 2016 [89]	F344	HCl	DP-SC (Dental pulp)	Bladder injection	2.0 × 10^6^	2–7	Anti-inflammatory
	/NSlc,						
	♀						
Stem Cells Dev 2015 [67]	SD	HCl	hUCB-MSC (Umbilical cord)	Bladder injection	1.0 × 10^6^	7	Wnt
	Rat						
	♀						
J Pineal Res 2014 [66]	SD	CYP	AD-MSCs (adipose tissue)	Intravenous tail injection	1.2 × 10^6^	3	Anti-inflammatory
	Rat						
	♂						

Chen et al. demonstrated that a combination of melatonin and AD-MSCs were superior to either alone in a cyclophosphamide-induced cystitis (CYP-IC) rat model due to synergistic antioxidant effects [66]. Song et al. evaluated the therapeutic effects of human umbilical cord blood MSCs (UCB-MSCs) in a hydrochloric acid-induced cystitis (HCL-IC) rat model and demonstrated that activation of the WNT signaling pathway alleviated IC/BPS [67]. Signaling pathways related to the sonic hedgehog (SHH) and WNT family genes and the associated epidermal (EGF), insulin-like (IGF), and fibroblast (FGF) growth factors were decreased in control HCL-IC bladders and increased in those receiving transplanted UCB-MSCs. Xiao et al. reported that beneficial effects of bone marrow MSCs (BM-MSCs) in protamine sulfate rat models were mediated by the TGF-β/MAPK signaling pathway associated with cell differentiation [68]. Xie et al. reported that UCB-MSC transplantation reduced inflammation, promoted proliferation, and inhibited apoptosis via activation of the AKT/mTOR signaling pathway in a CYP-IC rat model [69].

Choo’s group injected human embryonic stem cell-derived multipotent stem cells (hES-MSCs; MMSCs) in an HCL-IC model to see if the limited engraftment and survival capabilities of their counterparts from other tissues such as umbilical cord blood, bone marrow, and adipose tissue, were ameliorated, and whether the hES-MSCs could directly engage in repair of damaged tissues. MMSCs showed greater efficacy than adult BM-MSCs, with a therapeutic effect that persisted for up to 4 weeks after injection [70]. The WNT signaling pathway was also activated by MMSCs, and these stem cells directly differentiated into urothelium or pericytes of vascular endothelial tissue. GFP-tagged MMSCs were observed in rat bladders up to 6 months after injection. Micro-PET-MRI was performed to evaluate tumorigenicity immediately post-injection and 3, 6, 9, and 12 months afterward, and rats were sacrificed for necropsy after their last image work-up. These necropsies showed that the MMSCs did not migrate to other organs, and no signs of tumorigenesis were observed [70].

The same group also generated a ketamine-induced cystitis (KC) rat model that presented with similar clinical symptoms as the IC models described earlier [71–73]. Eight-week-old female rats were intravenously injected with ketamine (25 mg/kg) twice per week for three consecutive weeks. The control KC rats demonstrated bladder dysfunction, urothelial denudation, severe inflammation, mast cell infiltration, and increased apoptosis, similar to human IC/BPS patients. MMSCs were directly injected into the bladder one time, and histological and functional abnormalities were shown to be alleviated one week later [72]. The purported therapeutic mechanism of MMSCs in the KC model was anti-fibrosis [71, 72].

A limitation of the HCL-IC model is the acute nature of the urothelial damage caused; in humans, IC/BPS is a chronic disease. To generate an IC model with symptoms of a chronic nature, Choo and colleagues used an LPS-IC model to mimic chronic IC/BPS by instillation with protamine sulfate (PS) and lipopolysaccharide (LPS) once weekly for five weeks in 8-week-old female rats [74, 75]. Injection of MMSCs also significantly alleviated functional and histological damages in this LPS-IC model [74]. MMSC treatment was superior to that with BM-MSCs in the LPS-IC model, and the transplantation and differentiation of MMSCs were improved by SHH-WNT signaling pathway-activated growth factors including EGF, IGF, and FGF. High resolution intravital imaging has been employed to longitudinally observe in vivo activity of transplanted MMSCs in living animals. MMSCs-transplanted bladder tissue was monitored by a combination of confocal imaging of the external bladder wall and 1 mm micro-endoscopy of the internal bladder wall to evaluate the distribution, migration, and integration of transplanted MMSCs. The combination of longitudinal intravital confocal fluorescence imaging and microcystoscopy in living animals, together with immunofluorescence analyses of bladder tissues, have demonstrated that transplanted MMSCs differentiate into multiple cell types and gradually integrate into a perivascular-like structure up to 30 days after transplantation [74]. These reports from the Choo group provide the first evidence for improved therapeutic efficacy, long-term safety, and in vivo distribution and cellular properties of hESC derivatives in preclinical models of IC/BPS [70, 74].

## Conclusions

The efficacy and safety of stem cell therapy for bladder dysfunction has been shown in many preclinical models. However, only one pilot study in a single DUA patient has been conducted, and data from clinical trials are only available for a small number of SUI patients. Stem cell therapy evidence in the treatment of SUI is inconclusive, and indications for stem cell therapy for SUI have yet to be established. This limitation may be due to stem cell function loss following ex vivo expansion, poor in vivo engraftment or survival after transplantation, or a lack of understanding of the precise mechanisms of action underlying therapeutic outcomes and in vivo behavior of stem cells administered to target organs.

Several factors need to be addressed before the successful clinical application of stem cell therapy for bladder dysfunction. First, the pathophysiology of the target disease must be robustly investigated, as labelling the disease as idiopathic hinders comprehensive understanding of its underlying mechanisms. Second, the mode of action in stem cell therapy needs to be more clearly defined at the molecular level. Some preclinical studies only reported therapeutic effects as determined by histological and functional evaluations. Third, the optimal source, dosage, and route of stem cell injection, which are closely associated with safety issues, must be established and confirmed. This will require a large population prospective study with long-term follow-up despite the heterogeneous prevalence of bladder dysfunction. Finally, efficacy must be maximized to minimize the number of stem cells necessary for treatment. Reported therapeutic effects of stem cells have been proportional to the injected dosage, but higher concentrations of these cells carry an increased risk of side effects, including malignancy. For this reason, several studies have attempted to use adjuvant substances such as melatonin or external stimulation such as electrical stimulation and shock wave lithotripsy to enhance efficacy while reducing stem cell dosage.

Many research into stem cell therapy for bladder dysfunction remains on-going. Most preclinical studies have reported favorable therapeutic efficacy of stem cells regardless of injection route. However, animals and humans are not the same, and responses to the injections might vary across species. Results from the animal studies should thus be interpreted with caution when being evaluated for application in human patients. In particular, the optimization of stem cell sources targeted to the different disorders, and critical assessments of stem cell therapy safety are necessary to translate promising pre-clinical studies into clinical practice. Finally, comprehensive comparisons between stem cell-based approaches and existing therapies are a prerequisite to rendering stem cell treatment as the predominant strategy for treating intractable urological disorders in the future.
